# Detection of Microorganisms in Body Fluids via MTT-PMS Assay

**DOI:** 10.3390/diagnostics12010046

**Published:** 2021-12-27

**Authors:** Cheng-Han Chen, Yu-Ting Tsao, Po-Ting Yeh, Yu-Hsiang Liao, Yi-Tzu Lee, Wan-Ting Liao, Yung-Chih Wang, Ching-Fen Shen, Chao-Min Cheng

**Affiliations:** 1 Institute of Biomedical Engineering, National Tsing Hua University, Hsinchu 30013, Taiwan; gdc123.tw@gmail.com (C.-H.C.); sherry450047@gmail.com (Y.-T.T.); a29248510@hotmail.com (Y.-H.L.); u104015025@cmu.edu.tw (W.-T.L.); 2 Department of Emergency Medicine, Taipei Veterans General Hospital, Taipei 11217, Taiwan; s851009@yahoo.com.tw; 3 School of Medicine, National Yang Ming Chiao Tung University, Taipei 11221, Taiwan; 4 Department of Ophthalmology, National Taiwan University Hospital, Taipei 10002, Taiwan; ptyeh67@gmail.com; 5 National Defense Medical Center, Division of Infectious Diseases and Tropical Medicine, Department of Internal Medicine, Tri-Service General Hospital, Taipei 11490, Taiwan; wysywyst@gmail.com; 6 Department of Pediatrics, National Cheng Kung University Hospital, College of Medicine, National Cheng Kung University, Tainan 70101, Taiwan

**Keywords:** MTT-PMS assay, bacterial detection, point-of-care testing, infectious diseases, human body fluids

## Abstract

Early detection of microorganisms is essential for the management of infectious diseases. However, this is challenging, as traditional culture methods are labor-intensive and time-consuming. The 3-(4,5-dimethylthiazol-2-yl)-2,5-diphenyltetrazolium bromide-phenazine methosulfate (MTT-PMS) assay has been used to evaluate the metabolic activity in live cells and can thus be used for detecting living microorganisms. With the addition of NaOH and Tris-EDTA, the same approach can be accelerated (within 15 min) and used for the quick detection of common bacterial pathogens. The assay results can be evaluated colorimetrically or semi-quantitatively. Here, the quick detection by MTT-PMS assay was further investigated. The assay had a detection limit of approximately 10^4^ CFU/mL. In clinical evaluations, we used the MTT-PMS assay to detect clinical samples and bacteriuria (>10^5^ CFU/mL). The negative predictive value of the MTT-PMS assay for determining bacteriuria was 79.59% but was 100% when the interference of abnormal blood was excluded. Thus, the MTT-PMS assay might be a potential “rule-out” tool for bacterial detection in clinical samples, at a cost of approximately USD 1 per test. Owing to its low cost, rapid results, and easy-to-use characteristics, the MTT-PMS assay may be a potential tool for microorganism detection.

## 1. Introduction

Infectious diseases remain a major cause of ill health and socioeconomic burden in contemporary healthcare systems [1]. In 2017, 48.9 million cases of sepsis and 11 million sepsis-related deaths were reported worldwide [1]. According to the World Health Organization, infectious diseases are responsible for 54.4% of all global deaths. In particular, developing areas with low healthcare accessibility, such as Africa and Latin America, exhibit high incidence of infectious diseases and related mortality [1,2]. Despite developments in modern medicine, there are over 100,000 deaths attributed annually to infectious diseases in the United States [3]. Mortality risk from severe infection, or sepsis, increases by 7.6% for every hour of delayed antibiotic treatment; yet misuse or overuse of broad-spectrum empirical antibiotics leads to high rates of antimicrobial resistance [4,5]. By 2050, antimicrobial resistance-related mortality is estimated to increase to 10 million deaths per year, an outcome that will increase individual and communal economic burdens [5]. Furthermore, diagnostic delays and inaccuracies make patients more vulnerable to severe complications [6,7,8,9]. To effectively treat or manage infectious diseases, it is crucial to develop a tool for the early and precise determination of causal pathogens [2].

The process of infectious pathogen identification is comprising three main steps, i.e., pathogen detection, identification, and antimicrobial susceptibility testing [10]. When using conventional methods for pathogen identification, the results of incubation, culture, and staining are usually not available until 24 h after sample collection [5,11,12]. Microorganism culture, the long-term gold standard technique for microorganism identification, is labor-intensive and time-consuming [4,5]. These techniques have been widely utilized for microorganism detection since the 19th century [12,13,14], but they require at least 1–3 days for the initial incubation and approximately 1 week to provide confirmed antimicrobial susceptibility testing results [12,15].

Recently, biotechnological approaches have been introduced to accelerate the process of pathogen identification, including polymerase chain reaction (PCR) amplification, matrix-assisted laser desorption ionization time-of-flight (MALDI-TOF) spectrometry, and enzyme-linked immunosorbent assays (ELISA) [16,17,18,19]. However, these methods require highly qualified personnel and expensive equipment [16]. These disadvantages impede the implementation of these biotechniques in developing countries where infectious diseases continuously threaten and burden healthcare systems. Simple and inexpensive pathogen detection methods should be developed for resource-limited areas [20,21,22]. A simple and easy-to-use screening tool for the early detection of the presence of a pathogen is warranted.

Point-of-care testing (POCT), an emerging healthcare system, is specifically designed as a portable, rapid, and easily accessible approach for providing patients and healthcare personnel with immediate results. Various POCT techniques, focused on early bacterial detection, identification, or antimicrobial susceptibility testing, can be used for sepsis care [10,23]. Thus, POCT expedites clinical decisions and facilitates pathogen-targeted disease monitoring and management. To facilitate sepsis management, POCT can be divided into two categories, i.e., pathogen information acquisition and host monitoring. Pathogen acquisition consists of three parts, i.e., pathogen detection, pathogen identification, and antibiotic susceptibility testing [10]. With this approach, diseases can be diagnosed at an early stage, resulting in improved outcomes for patients by triaging infectious patients and accelerating treatment [24,25].

**Figure 1:**
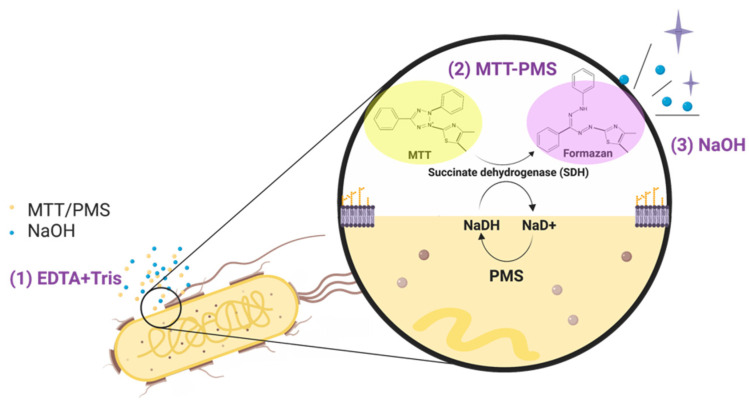
Mechanism of the 3-(4,5-dimethylthiazol-2-yl)-2,5-diphenyltetrazolium bromide-phenazine methosulfate (MTT-PMS) assay: (**1**) Tris/EDTA solution was used to penetrate bacterial cell walls and MTT/PMS was allowed to react with cellular succinate dehydrogenase; (**2**) MTT-PMS and NaOH were added to the solution. The lightly-colored tetrazolium salt was reduced to an intensely purple formazan form; (**3**) NaOH stimulated and amplified the reaction. The entire process was completed within 15 min. The figure was created using BioRender.com.

The 3-(4,5-dimethylthiazol-2-yl)-2,5-diphenyltetrazolium bromide-phenazine methosulfate (MTT-PMS) assay is a colorimetric technique that has been previously used to evaluate the metabolic activity of live cells [26]. In this approach, the lightly-colored tetrazolium salt is enzymatically reduced to its intensely purple-blue formazan form (Figure 1). The tetrazolium reduction reaction is associated with the electron transport system of viable microorganisms [26]. Although the mechanism of reduction of MTT in bacteria remains unknown, the quantity of reduced formazan is proportional to the number of viable bacteria in the test solution [26,27]. This reaction can be monitored spectrophotometrically, providing a semi-quantitative readout indicating the presence of microorganisms [26]. The traditional MTT assay usually requires several hours of processing time. The MTT-PMS assay is performed using an intermediate electron acceptor, PMS, which accelerates the reaction allowing for a turnaround time of under 45 min [28,29]. Our previous studies have demonstrated that the MTT-PMS assay can provide a rapid and semi-quantitative result of bacterial detection in water [16]. With the addition of Tris-EDTA (Tris-Ethylenediaminetetraacetic acid) and sodium hydroxide, the presence of bacteria in water was successfully detected within 15 min [16]. The quick turn-around time for this test lends to the potential application of the MTT-PMS assay for detecting the presence of bacteria in clinical human body fluids and as a POCT platform to screen for pathogen presence. However, to the best of our knowledge, the efficacy of the MTT-PMS assay in clinical samples for bacterial detection remains unknown. Therefore, this study was designed to evaluate the effectiveness of the MTT-PMS assay for bacterial screening in a range of solutions from phosphate-buffered saline to clinical human body fluid samples.

## 2. Materials and Methods

### 2.1. Study Design

In this study we conducted MTT-PMS assays using three different conditions. First, to evaluate the detection efficacy and accuracy, we conducted the assays using varied concentrations of bacteria in PBS. Second, we simulated plasma conditions using intraocular fluid; a transparent, watery, plasma-like fluid with low protein concentration and few blood cells. We compared the results of the MTT-PMS assay with intraocular fluid to results from intraocular culturing tests from samples collected at National Taiwan University Hospital (a tertiary hospital with 2400 beds in Taiwan). Because of the blood-ocular barrier, intraocular samples present an uncomplicated background for bacterial detection in human body fluids [30]. Finally, we conducted MTT-PMS assays for bacteriuria detection in clinical urine samples collected from Taipei Veteran General Hospital (TVGH, a tertiary hospital with 2700 beds in Taiwan) and compared the results with those of routine urinary culture. Human urine samples are complex fluids with varying concentrations of inorganic salts and organic compounds, including hormones, proteins, and a wide range of metabolites [30]. The image-recording system used included a Tecan Sunrise™ Absorbance Microplate Reader (8708 Männedorf, Switzerland: Tecan) and an iPhone XS (Cupertino, CA, USA: Apple inc.). ImageJ software [31] was used for the image analysis. Furthermore, *Staphylococcus aureus* (TL341), *Escherichia coli* (DH5α), *Klebsiella pneumoniae* (ATCC 23357), and *Pseudomonas aeruginosa* (PA01) were used as target bacteria in this study. Bacterial viability was calculated using the following equation:

CFU (colony-forming units)/mL = (number of colonies × dilution factor) × 10(1)

More details regarding the reagents, equipment, and bacterial preparation are provided in the Supplementary Materials.

### 2.2. Reagents and Protocol Development

The MTT-PMS solution was prepared by adding 5 mg/mL MTT to 0.2 mg/mL PMS. The Tris/EDTA solution (pH 8.0) was prepared by diluting Tris-EDTA buffer to a concentration of 10-7 M EDTA with 0.1 mM Tris buffer. Tris/EDTA solution (30 μL) was added to a 30 μL urine sample and incubated at 25 °C for 5 min before adding 30 μL of the MTT-PMS solution, followed by the addition of 2 μL of NaOH. Colorimetric detection was performed at 595 nm using a plate reader. Tris/EDTA solution was used to break down bacterial cell walls and allow MTT-PMS to react with cellular succinate dehydrogenase. Succinate dehydrogenase transformed the yellow-colored MTT into purple formazan. PMS facilitated this reaction as an intermediate electron acceptor. In the last step, NaOH (0.1 N) was used to adjust the pH to amplify the colorimetric signal.

### 2.3. Patient Selection

#### 2.3.1. Intraocular Fluid

After obtaining approval from the Institutional Review Board (IRB) of the National Taiwan University Hospital (IRB number 1083707256), we obtained intraocular fluid samples from patients diagnosed with endophthalmitis just before their intravitreal antibiotic injection therapy. This study was conducted following all relevant guidelines and tenets of the Declaration of Helsinki. All patients recruited in this study provided written informed consent before enrollment. A 27-gauge needle was used to collect the intraocular fluid and the samples were immediately stored at 4 °C until further analysis.

#### 2.3.2. Urine Sample

After approval from the IRB of TVGH (IRB, TPEVGH 2019-10-001B), we obtained urine samples from patients admitted to the emergency department who were suspected of having a urinary tract infection (UTI). This study was conducted following all relevant guidelines and tenets of the Declaration of Helsinki. All patients recruited in this study provided written informed consent before enrollment. Routine examination of the urine samples was performed, and the samples were divided into three aliquots. One aliquot was checked with a urinary dipstick (MEDITAPE^TM^ UC-11A. Kobe, Japan: Sysmex Corp.) at TVGH. Another aliquot was sent to the TVGH Central Lab for routine urinary culture, the gold standard test (incubation at 37 °C for 24 h) with a positive test cutoff value of 10^5^ CFU/mL. The third aliquot was immediately stored at 4 °C and sent to the laboratory for further analysis.

### 2.4. Statistical Analysis

All MTT-PMS assay results are expressed as CFU/mL. For the microplate reader-based protocol, the optical intensity results measured by the microplate reader were expressed as the mean ± standard deviation. For the smartphone camera-based protocol, the images were converted into grayscale using ImageJ software with the formula:

gray = (red + green + blue)/3.(2)

Comparisons of the mean intensity between different bacterial concentrations were analyzed using the Student’s *t*-test for normally distributed data or the Mann–Whitney U-test for non-normally distributed data. The intra-assay coefficients of variability for the MTT-PMS assay were calculated by dividing the standard deviation of the blank value by the mean of the blank value and multiplying by 100. For all statistical results, a *p*-value < 0.05 was considered statistically significant. All statistical analyses were performed using IBM SPSS Statistics for Windows (version 22.0. Armonk, NY, USA: IBM Corp.).

## 3. Results

### 3.1. Development of MTT-PMS Bacterial Detection Assay

**Figure 2:**
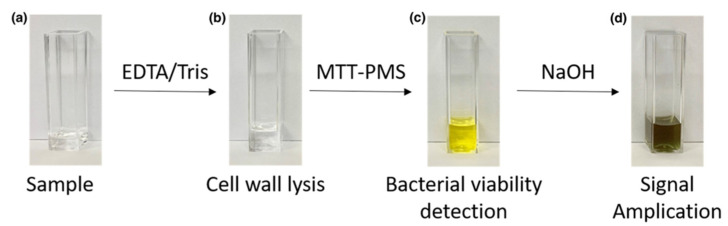
Sequential color changes associated with the 3-(4,5-dimethylthiazol-2-yl)-2,5-diphenyltetrazolium bromide-phenazine methosulfate (MTT-PMS) assay: (**a**) Bacterial samples were prepared using a buffer system; (**b**) EDTA/Tris was added for cell wall lysis. (**c**) MTT-PMS was added to the solution. The lightly-colored tetrazolium salt was reduced to an intensely purple formazan form. (**d**) NaOH was added for reaction amplification.

The sequential color changes at the different stages of the MTT-PMS assay are illustrated in Figure 2. To clearly demonstrate the colorimetric response, the volume of the reagents used in the experiments shown in Figure 2 were 10-fold higher than that typically used for the microplate protocol but were the same volumes which are used in the smartphone-based protocol. After precipitation of the purple-colored formazan with NaOH, the final brown/dark green solution was formed by mixing yellow PMS and purple formazan. The final colorimetric output was captured 5 min after the final reagent was added.

**Figure 3:**
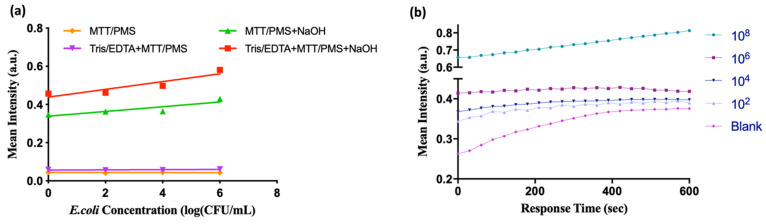
Establishment of the 3-(4,5-dimethylthiazol-2-yl)-2,5-diphenyltetrazolium bromide-phenazine methosulfate (MTT-PMS) bacterial detection assay protocol: (**a**) Functional validation of each reagent: The colorimetric results of MTT-PMS and Tris/EDTA + MTT-PMS assays demonstrated no differences in bacterial concentration at 5 min (purple and orange lines). In the group treated with MTT-PMS + NaOH, a semi-quantitative colorimetric signal was observed at 5 min (green line). With the addition of Tris-EDTA + MTT-PMS + NaOH, the quantitative colorimetric response became visible (red line). The slope of the green line is 1.6-fold higher than that of the blue line (0.02 vs. 0.012); (**b**) Dynamic colorimetric results of the MTT-PMS assay at 595 nm. Signals were recorded every 20 s for 10 min.

The effectiveness of each reagent was tested, and the results are shown in Figure 3. The colorimetric differences between low and high bacterial concentrations were evident when all three reagents, namely, MTT-PMS, Tris/EDTA, and NaOH solution, were added. All reagents were used in subsequent experiments. The optimized protocol recorded dynamic colorimetric results at 595 nm. The results indicated that the MTT-PMS biochemical reaction tended to remain stable after approximately 300 s (Figure 3b). Accordingly, we recorded all subsequent colorimetric results after 5 min of incubation. The method for establishing the standard curves is described in the Supplementary Materials.

**Figure 4:**
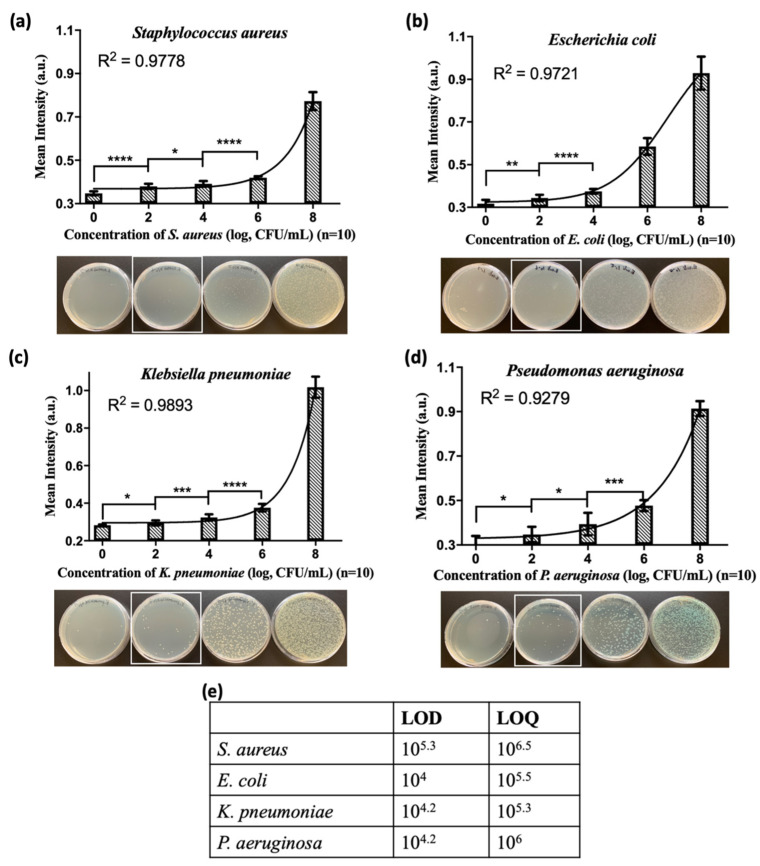
Detection capacity of the 3-(4,5-dimethylthiazol-2-yl)-2,5-diphenyltetrazolium bromide-phenazine methosulfate (MTT-PMS) assay for different bacterial species. The results were recorded using a microplate reader at 595 nm, and all experiments were repeated 10 times to ensure efficacy and reproducibility. Results of the MTT-PMS assay for: (**a**) *S. aureus*; (**b**) *E. coli*; (**c**) *K. pneumoniae*, and (**d**) *P. aeruginosa*. All assays demonstrated significant differences among the different bacterial concentrations. (* *p* < 0.05, ** *p* < 0.01, *** *p* < 0.001, **** *p* < 0.0001); (**e**) Limit of detection (LOD) and limit of quantification (LOQ) of the MTT-PMS assay in a buffer system for different species as calculated using Hill’s equation).

**Figure 5:**
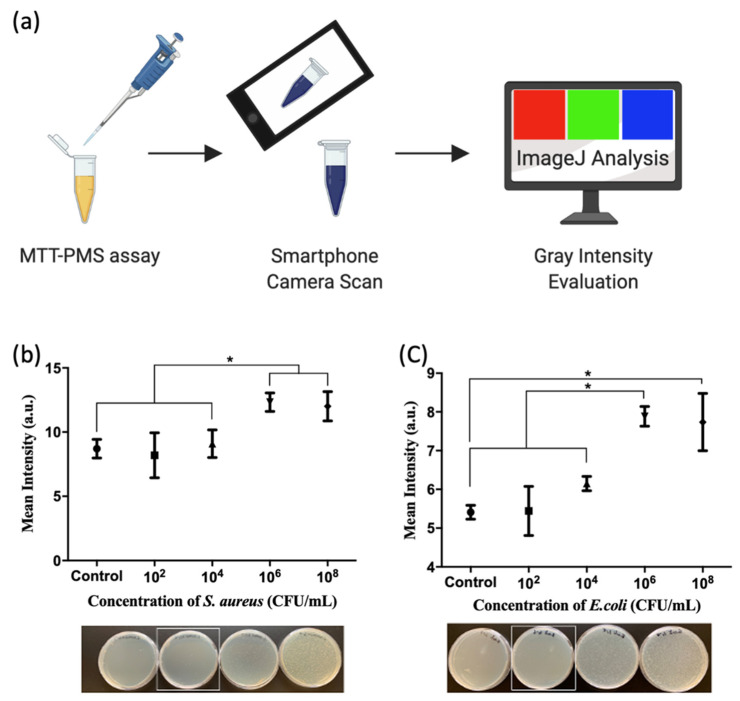
Integration of the 3-(4,5-dimethylthiazol-2-yl)-2,5-diphenyltetrazolium bromide-phenazine methosulfate (MTT-PMS) assay with a smartphone camera: (**a**) After adding MTT-PMS to the experimental tube, results were scanned with a smartphone (iPhone XS, Apple). The gray intensity was evaluated using a freeware App “ImageJ”[31]; Results of the MTT-PMS assay for (**b**) *S. aureus* and (**c**) *E. coli*. Figure created in BioRender.com. * *p* < 0.05.

We used the MTT-PMS assay to detect *S. aureus*, *E. coli*, *P. aeruginosa*, and *K. pneumoniae* in a buffer system. The results are shown in Figure 4. The MTT-PMS assay demonstrated the ability to detect all four bacterial species in the blank, up to a concentration of 10^8^ CFU/mL. The results were obtained using a microplate reader at 595 nm with methods calibrated for bacterial viability of each individual species of bacteria. The results of the spread plate method were compared with those of the MTT-PMS assay, and this is shown in Figure 4. Figure 4e shows the limit of detection and limit of quantification of the MTT-PMS assay for different species calculated using Hill’s equation. The applicability of the MTT-PMS assay to a smartphone camera-based protocol was also assessed using *S. aureus* and *E. coli* as Gram-positive and Gram-negative species, respectively. The results are shown in Figure 5. Using the smartphone camera-based protocol, the MTT-PMS assay successfully distinguished bacterial concentrations below 10^4^ CFU/mL and above 10^6^ CFU/mL, calculated using Hill’s equation.

### 3.2. Clinical Evaluation of MTT-PMS Assay for Living Microorganism Detection

#### 3.2.1. MTT-PMS Assay of Human Intraocular Fluid

Six patients diagnosed with endophthalmitis were enrolled in this study. Group 1 comprised five samples obtained before the patients received any treatment. Group 2 comprised one sample obtained from the remaining patient after the administration of an intra-vitreous antibiotic injection. Of the six patients, five were diagnosed with exogenous endophthalmitis and one was diagnosed with endophthalmitis. Three aqueous humor samples and three vitreous humor samples were acquired. The final diagnosis was confirmed using a hospital report by combining clinical findings (symptoms, signs, and response to antibiotics) and bacterial culture reports. The basic characteristics of the six samples are listed in Table 1.

#### 3.2.2. MTT-PMS Assay of Human Urine Samples

A total of 116 patients were included in the analysis (Table S1); most of them were females (71%), with a mean age of 73.5 years. Urine culture results of >10^5^ CFU/mL were considered positive and the threshold of our MTT-PMS assay was >10^5^ CFU/mL; however, sensitivity and specificity were 70% and 44.19%, respectively (Table 2a). As the spectrophotometric analysis might have been affected by the hemoglobin or abnormal blood in the urine samples, we excluded samples with positive abnormal blood (OB, *n* = 59) and performed a subgroup analysis (OB group, Table 2). The sensitivity and specificity of the MTT-PMS assay in the subgroup were 100% and 44.00%, respectively.

## 4. Discussion

In this study, we modified the transitional MTT assay, extending its use to directly screen live bacteria in clinical human body fluid samples. MTT is a well-known colorimetric reagent for assessing metabolic activity within cells and has been utilized for a variety of experimental purposes, such as mycoplasma screening, detection of superoxide radicals, microbial viability, and growth estimation [26]. Although the exact mechanism of MTT reduction in bacteria is unclear, the MTT assay is widely utilized for detecting bacterial respiratory activity and viability [26,32,33]. The MTT assay traditionally takes several hours to complete [12]. We modified the traditional MTT assay to create an MTT-PMS assay, which introduces an additional electron-acceptor, with several optimized aspects including improved bacterial wall penetration and signal enhancement. Tris/EDTA solution was added to improve the bacterial wall penetration. The metal chelating agent, EDTA, can bind to divalent cations, such as Ca^2+^ and Mg^2+^, on the cell wall and increase cell permeability [34,35]. It can also specifically loosen the lipopolysaccharides of Gram-negative bacteria [36]. The pH was maintained at 8 to achieve maximal permeability. PMS solution was added to our MTT assay to accelerate the reaction between MTT and the dehydrogenase system present in the bacterial cytoplasmic membrane [37]. The inclusion of an intermediate electron acceptor facilitated the MTT reduction reaction [28,29]; 100 mM NaOH (0.1 N) was added to create an alkaline environment to accelerate the reaction and enhance the signal [38,39]. By combining each of these reagents and using them to augment our assay processes, the entire MTT-PMS assay for bacterial screening could be completed within 15 min [16]. The formazan crystals were stably dissolved in an alkaline solvent thus our solution was maintained at pH 8 for stable dissolution [40]. The optimal wavelength of the formazan solution varies from 550 nm to 600 nm, depending on the selected solvent [26,40]. In the MTT-PMS assay, our previous experience showed that the optimal wavelength was 595 nm for screening viable bacteria in water [16].

Using PBS as a standard condition, the standard curve was calculated using Hill’s equation. The limit of detection was 10^4^–10^5.3^ and the limit of quantification was 10^5.3^–10^6.5^ (Figure 4e). These results are comparable to those generated by the current technology. Current bacterial detection systems detect pathogens through CO_2_ emission, based on the colorimetric detection of pH changes caused by the CO_2_ output of pathogenic microorganisms and have been successfully used in clinical settings since 1990 [41]. The reported threshold concentration for detection using these systems is approximately 10^4^ CFU/mL [11], and the processing time is approximately 10–15 h. More importantly, to execute these techniques, a central laboratory and trained technicians are required [41,42]. For the broader application of the MTT-PMS assay, we developed a colorimetric MTT-PMS assay using ImageJ, an open-source image processing program, using a smartphone (iPhone XS, Apple). The semi-quantitative results produced by integrating smartphone-based analysis with MTT-PMS assay results allowed us to differentiate bacterial concentrations from 10^4^ to 10^6^ CFU (Figure 5). The addition of smartphone technology may expand the utility of the MTT-PMS assay for bacterial screening.

This study further evaluated the clinical efficacy of the MTT-PMS assay for bacterial screening to determine whether it could provide semi-quantitative results when differentiating bacterial concentrations range from 10^0^ CFU/mL (sterile conditions) to 10^8^ CFU/mL. Two types of clinical samples were selected for this study: intraocular fluid and urine samples. The intraocular fluid is a transparent and sterile fluid, similar to plasma [43]. Although intraocular sample collection is rare and laborious, low-protein-containing intraocular fluids represent a relatively simple situation for analysis. In contrast, urinary tract infections are highly prevalent diseases and bacteria-contaminated urine samples are more accessible [44], however, urine samples are challenging because they are 90% water and may include other minor organic or inorganic materials depending on host’s metabolic conditions [45].

Intraocular fluid is sterile, but microorganisms, such as bacteria or fungi, may infect the eye and cause endophthalmitis, an acute vision-threatening condition. Delayed diagnosis or inappropriate treatment of such diseases may result in catastrophic consequences, including severely impaired visual acuity within 12–24 h [46,47,48]. Endophthalmitis is diagnosed based on either clinical judgment or through microbiological culturing of samples obtained through vitreous or aqueous aspiration [49]. Due to endophthalmitis being rare and sample aspiration being an invasive procedure, only six intraocular fluid samples were obtained for this study. We compared the correlation between the MTT-PMS assay and a definitive diagnosis (either a culturing test or clinical judgment) (Table 1). Five samples in Group 1 were collected before the antibiotic treatment and all the samples produced a positive colorimetric MTT-PMS reaction. These results were consistent with a definitive diagnosis of endophthalmitis. Positive MTT-PMS assay results indicated the presence of microorganisms in the aqueous sample. Microorganism culture might have been hindered by the presence of fastidious organisms, prior antimicrobial exposure, or bacterial concentrations that were too low for culture being the outcome [50,51]. The sample in Group 2 was collected after intra-vitreous antibiotic administration, and therefore, neither the MTT-PMS assay nor the respective vitreous culture was positive. These consistent MTT-PMS assays and vitreous culture results imply that there were no live microorganisms in the sample.

UTI is a common infectious disease with a 50–60% lifetime incidence among adult women and a 10% incidence in the entire population [44]. Moreover, UTI accounts for the second most common nosocomial infection and can cause sepsis or septic shock if left untreated [52]. The diagnosis of UTI is based on clinical presentation and bacteriuria confirmed by a positive urine culture result. Bacteriuria was defined as a bacterial culture of >10^5^ CFU/mL. Although urine culture is the gold standard for bacteriuria diagnosis, it requires several days [52]. Therefore, we introduced an MTT-PMS assay approach for the real-time screening of bacterial presence in urine samples. Our MTT-PMS assay demonstrated a negative predictive value (NPV) of 79.59% (Table 2). The exact mechanism remains unclear, but previous studies have reported that the presence of heme might interfere with the spectral absorption and the redox reaction of the MTT-PMS assay, causing either false-positive or false-negative results [53,54,55]. Hence, we excluded urine samples that contained abnormal blood. The results showed that the NPV of the MTT-PMS assay was 100% when urine samples with abnormal blood were excluded (Table 2). With its high sensitivity and high NPV, the MTT-PMS assay may be a useful bacteriuria screening tool for urine samples that do not contain abnormal blood. Negative MTT-PMS results indicated a low risk of bacteriuria.

A point-of-care testing device should be easy-to-use, accurate, cost-efficient, rapid, and require only a non-technical user to operate, detecting targeted objects with limited infrastructure [56]. Our previous study reported that the MTT-PMS assay is a rapid detection tool for viable bacteria in water [16]. This study of the MTT-PMS assay in clinical samples demonstrated high sensitivity for detecting microorganisms in both intraocular fluid and urine. Compared to contemporary bacterial detection methods, the spread-plate or multi-tube fermentation requires a few days to obtain the results and the real-time quantitative PCR or ELISA requires several hours [16,57,58,59]. In contrast, this MTT-PMS assay requires only a small sample size (30 μL), is inexpensive (approximately USD 1 per test), and requires only 15 min to obtain semi-quantitative results [16].

A point-of-care testing device can only make a difference if this technique can be commercialized. For commercial purposes, this technique should be integrated with disposable or microfluidic platforms, implement surface-bound detect techniques, and rely on open-source hardware or software [56]. Using a colorimetric device, we can evaluate the results of the MTT-PMS assay easily and accurately [16]. In addition to being readable using a microplate reader, this study further demonstrates the efficacy of a semi-quantitative, colorimetric analysis of the MTT-PMS assay using an open-source application, ImageJ (Figure 4). We previously successfully integrated the MTT assay into a low-cost, laboratory-free, easy-to-use, paper-based device to evaluate porcine sperm motility. The MTT assay was embedded on a designed paper-based device, and the colorimetric results could be evaluated via a smartphone application [60,61,62,63]. The integration of this technique into a paper-based device that can be analyzed with a smartphone makes it highly portable and accessible for use in local clinics and communities. The collective characteristics of the MTT-PMS assay and associated point-of-care integration indicates that it might be a useful “rule-out” tool for pathogen presence screening that could be used before routine culture techniques.

This study evaluated a specific and novel diagnostic application of the MTT-PMS assay, but it has several limitations. First, the sample size was limited because of the low prevalence of intraocular infection and the highly invasive nature of intraocular fluid aspiration. Moreover, because the contents of human urine are heterogeneous and variable according to patient metabolic conditions, it was challenging to develop a “standard” condition to calculate the limit of detection in the urine sample. Furthermore, the interference of heme or hemoglobin hindered the utility of the MTT-PMS assay. Additional studies are required to eliminate heme interference and broaden the utility of the MTT-PMS assay, especially for use with samples containing blood. Finally, the limit of detection of the MTT-PMS assay was only 10^4^–10^5.3^, a value that should be improved by further optimizing the elements of the MTT-PMS assay.

In conclusion, our study revealed that the MTT-PMS assay has several potential uses and advantages:

1. It could be used as a “rule-out” test for infectious diseases screening. A negative MTT-PMS assay result may indicate a low possibility of “live” microorganism presence [26,64]. Using screening methods before conventional microorganism culture may reduce the potential risk of stratification of febrile patients and guide initial treatment [23].
2. Rapid results. By adding reagents to improve bacterial wall penetration, facilitating the provision of an additional electron acceptor, and enhancing the resulting signal, this MTT-PMS assay has been optimized to quickly detect the presence of microorganisms and provide real-time semi-quantitative screening of infectious diseases within 15 min.
3. Semi-quantitative readout. This process provides rapid and semi-quantitative information regarding the presence of microorganisms without sophisticated equipment or trained technicians.
4. Potential POCT device. This MTT-PMS assay may be integrated into a paper-based device and colorimetric analysis can be performed using a smartphone color-recognition application [16,60,61,62,63]. Moreover, the MTT-PMS assay can further be embedded into a disposable paper-based device and evaluated with a smartphone or an open-source application (Image J). These features indicate the MTT-PMS assay has the potential to be commercialized.

## 5. Conclusions

Infectious diseases impose a huge burden on both healthcare and socio-economic systems; thus, real-time accurate diagnostic methods are required [1,4,25]. The MTT-PMS assay provides a low-cost, rapid, and easy-to-use approach for detecting the presence of “live” microorganisms [65], and therefore possess the potential to be commercialized. It can be incorporated into conventional microorganism culture methods as a rapid infectious disease screening tool. Although we found a high NPV for the urinary MTT-PMS assay, it is unclear how abnormal blood interferes with the MTT-PMS assay. Further investigation may reveal the broader applicability of MTT-PMS assay-based microorganism detection.

## Figures and Tables

**Table 1:** Characteristics of patients with endophthalmitis and their test results.

	No.	Age	Sex	Eye Site	Disease Diagnosis	Underlying Disease	Sample Type	Culture Result	MTT-PMS Assay Results
Group 1	1	62	F	OD *	Endophthalmitis (exogenous)	Right breast cancer	Aqueous	G(+) ^‡^ cocci 1+ ^‡‡^	10^4^–10^6^
	2	62	F	OD	Endophthalmitis (exogenous)	Right breast cancer	Vitreous	G(+) ^‡^ cocci 1+	10^6^–10^8^
	3	62	F	OD	Endophthalmitis (exogenous)	Right breast cancer	Aqueous	N/A	10^6^–10^8^
	4	47	F	OD	Endophthalmitis (endogenous)	Sepsis, Right breast cancer	Aqueous	Aqueous: no anaerobic pathogen. ^†^	10^6^–10^8^
	5	73	F	OS *	Endophthalmitis (exogenous)	Diabetes mellitus, Hypertension	Vitreous	*Candida parapsilosis* complex 1+	>10^8^
Group 2 **	1	68	M	OS	Endophthalmitis (exogenous)	Hypertension, ESRD with HD	Vitreous	No growth	0

* OD: oculus dexter, right eye; OS: oculus sinister, left eye; ** Group2: After antibiotic treatment; ^‡^ G denotes “Gram stain,” G (+) Gram stain positive species, G (−) Gram stain negative species; ^‡‡^ The semi-quantitative scoring of Gram stain was based on the number of bacteria per high-power (×1000) oil immersion field: 0 = no bacteria per field; 1+ = less than one bacterium per field; 2+ = 1–5 bacteria per field; 3+ = 6–30 bacteria per field, and 4+ = more than 30 bacteria per field 1 [1]; † Although the final aqueous culture was negative, the patient’s urine culture showed Enterococcus faecium >100,000, and the sputum culture showed G (−) cocci 1+. The final diagnosis made by ophthalmologists was endophthalmitis.

**Table 2:** Comparison of routine urine culture reports and 3-(4,5-dimethylthiazol-2-yl)-2,5-diphenyltetrazolium bromide-phenazine methosulfate (MTT-PMS) assay results (cut-off value urine culture >105 CFU/mL).

**Comparison of Urine Culture Report >10^5^ CFU/mL, *n* = 116**				
		Urine Culture >10^5^ CFU/mL (*n*)		
		Positive	Negative	Total
MTT-PMS assay 10^5^ (*n*)	Positive	20	47	67
	Negative	10	39	49
	Total	30	86	116
Sensitivity	Specificity	Accuracy	PPV *	NPV **
66.67%	45.35%	50.86%	29.85%	79.59%
**Comparison of Urine Culture Report >10^5^ CFU/mL, *n* = 59, OB– ^†^**				
		Urine Culture >10^5^ CFU/mL (*n*)		
		Positive	Negative	Total
MTT-PMS assay10^5^ (*n*)	Positive	9	28	37
	Negative	0	22	22
	Total	9	50	59
**Sensitivity**	**Specificity**	**Accuracy**	PPV *	NPV **
100.00%	44.00%	52.54%	24.32%	100.00%

* PPV: positive predictive value; ** NPV: negative predictive value; ^†^ OB–: negative for abnormal blood; (a) Comparison of the MTT-PMS assay with urine culture results. The sensitivity, specificity, PPV, and NPV were 66.67%, 45.35%, 29.85%, and 79.59%, respectively; (b) Comparison of the MTT-PMS assay results with urine culture reports in urine without abnormal blood. The sensitivity, specificity, PPV, and NPV were 100%, 44%, 24.32%, and 100%, respectively.

## Data Availability

The datasets of this research are available on request to the corresponding author.

## Supplementary Materials

The supplementary material is available online at https://www.mdpi.com/article/10.3390/diagnostics12010046/s1. Details of the reagents, equipment, bacterial preparation, and establishment of standard curves are shown in Table S1. Characteristics and results of patients with urinary tract infections are also shown in the Supplementary Materials.
