# Supplementary material for: Detection of Microorganisms in Body Fluids via MTT-PMS Assay
Source: Diagnostics (Basel). 2021 Dec 27;12(1):46. doi: 10.3390/diagnostics12010046 (PMC8774610; doi:10.3390/diagnostics12010046)
Supplement: Supplementary file 1 [file diagnostics-12-00046-s001.zip › diagnostics-1495979-supplementary.pdf]

Supplementary information

Detection of microorganisms in body fluids *via* MTT-PMS assay

1. Details of reagents, equipment, bacteria preparation, and the generation of standard curves

1.1. Reagents and Equipment

We purchased 3-(4,5-dimethylthiazol-2-yl)-2,5-diphenyltetrazolium bromide (MTT) and phenazine methosulfate (PMS) from Sigma-Aldrich (Cat No.: M2128 and P9625, St. Louis, USA, respectively). Tris/EDTA solution was prepared by diluting 10X Tris/EDTA buffer (purchased from GMbiolab; Cat No.: GB06-1) with Tris solution (Sigma-Aldrich; Cat No.: T1503). The pH of the Tris solution was adjusted using HCl and NaOH. NaOH was purchased from SHOWA Chemicals (Cat No. 1943–0160). Phosphate-buffered saline (PBS) was purchased from Sigma-Aldrich (Cat. No. P4417). The image-recording system used a Tecan Sunrise™ Absorbance Microplate Reader and an iPhone XS (Apple). ImageJ used for image analysis was downloaded from http://rsbweb.nih.gov/ij/download.html. The Type A2 biological safety cabinet (Thermo Scientific™ 1300 Series Class II and Type A2) and biological safety cabinet packages were used.

1.2. Bacterial Species

Staphylococcus aureus (TL341), *Escherichia coli* (DH5α), *Klebsiella pneumoniae* (ATCC 23357), and *Pseudomonas aeruginosa* (PA01) were used in this study. The bacterial species were isolated using the streak plate method. Single colonies were inoculated into 3 mL of fresh Luria-Bertani (LB) medium and cultured at 37 °C for 18 h before use. Bacterial viability was verified using the spread plate method. Briefly, 100 μL of the 10-fold serially diluted bacterial solution was spread onto an LB agar plate and the inoculated plate was incubated overnight at 37 °C. Plates with 30–300 colonies were selected to calculate the bacterial viability using the following equation:

$$\text{CFU (colony-forming units) / mL} = (\text{number of colonies} \times \text{dilution factor}) \times 10 \quad (1)$$

1.3. Establishment of Standard Curves

The MTT-PMS assay was used to detect bacterial viability as follows: The cultures grown in LB were centrifuged and the resulting pellet was resuspended in PBS. To generate a standard curve, the bacterial solution was diluted with PBS to prepare solutions with four different concentrations, i.e., 1×10^8, 1×10^6, 1×10^4, and 1×10^2 CFU/mL. To expand the utility of the assay, gram-positive *S. aureus* and gram-negative *E. coli*, *P. aeruginosa*, and *K. pneumoniae* species were selected for testing. Serially diluted bacterial or clinical samples (30 μL) were added to a 96-well plate followed by the addition of 30 μL of a cell-wall penetrating agent (Tris/EDTA). After incubation for 5 min, 30 μL of the MTT-PMS mixture was added to assess the bacterial viability. Finally, 2 μL of 100 mM NaOH was added to accelerate the reaction and enhance the colorimetric signal. After another 5 min of incubation, the plate was scanned at 595 nm to generate standard curve data. All procedures were conducted in a Type A2 biological safety cabinet to minimize unwanted contaminants, such as dust or environmental microorganisms, and the experiments were conducted in a biosafety level 2 laboratory. Each assay cycle was completed within 15 min, at a cost of approximately 1 USD.

1.4. Use of a Simple Smartphone Protocol to Evaluate the Assay

For this experiment, *S. aureus*, *E. coli*, *P. aeruginosa*, and *K. pneumoniae* were selected as model organisms. The reagents used in this experiment were added to plastic cuvettes in a volume 10-fold higher than that for a typical microplate reader–based protocol. For this, 300 μL of the serially diluted bacterial solution, Tris/EDTA buffer, MTT-PMS reagents, and 20 μL of NaOH were added sequentially according to the same protocol and ratios mentioned above. The colorimetric results were recorded using a simple smartphone camera and analyzed using ImageJ. These experiments were conducted on a normal bench rather than a biosafety level 2 laboratory to simulate ill-equipped resource-limited conditions.

Table S1. Characteristics and results of patients with urinary tract infections.

**Demographic characteristics (*n* = 116)**	
Age, median (IQR), years	73.5 (58 - 81.75)
Female sex, Number (%)	71 (61.21)
GFR† (mL/min/1.73 m^2), Median (IQR)	75 (39–96.85)
Bed-ridden status, Number (%)*	41 (35.34)
Urinary catheter, Number (%)	27 (23.28)
Healthcare-associated infection, Number (%) ‡	39 (33.62)
Comorbid conditions, Number (%)	
Chronic obstructive pulmonary disease	9 (7.76)
Chronic kidney disease (GFR < 30)	23 (19.83)
Type 2 diabetes mellitus	34 (29.31)
Hypertension	57 (49.14)
Coronary artery disease	30 (25.86)
Congestive heart failure	24 (20.69)
Cerebrovascular accident	24 (20.69)
Autoimmune disease	5 (4.31)
Malignancy history	39 (33.62)
Chemotherapy in recent 2 weeks	8 (6.90)
Recent surgery in recent 1 months	11 (9.48)
Urolithiasis	7 (6.03)
Benign prostate hypertrophy	18 (15.52)

*Bed-ridden status is defined as patients who are not ambulatory and require total assistance for daily care; †GFR, glomerular filtration rate; GFR < 60 mL/min/1.73 m^2 indicates impaired kidney function [1]; ‡ Healthcare-associated infection [2] is defined as an infectious disease contracted in a hospital or other healthcare facility that first appeared in 48 h or more after hospital admission or within 30 days after receiving medical help.

References

1. Chapter 1. Definition and Classification of CKD. Kidney Int. Suppl. 2011. 2013, 3 (1), 19–62.
2. Haque, M.; Sartelli, M.; McKimm, J.; Abu Bakar, M. Health Care-Associated Infections - an Overview. Infect. Drug Resist. 2018, 11, 2321–2333.
